# On Diseases of the Liver and Its Appendages

**Published:** 1831

**Authors:** 


### VIII.

### ON DISEASES OF THE LIVER AND ITS APPENDAGES. By *M. Andral.*

[Pathological Anatomy, Vol. II.]

IN the 28th No. of this journal, we selected an article from this able work, now rendered accessible to English readers by the excellent translation of Drs. Townsend and West, as a specimen of the performance. We now proceed to give an abstract of that chapter which treats of the pathological anatomy of the liver.

The diseases of this organ, M. Andral observes, are seated either in the substance or the excretory ducts. He therefore treats of these separately.

### I. DISEASES OF THE PARENCHYMA OF THE LIVER.

"If we carefully examine (says he) the structure of the liver, we find that it is composed of two substances; one reddish, formed chiefly by the ramifications of the capillary vessels of the organ; and the other white or yellowish, which seems chiefly destined for the secretion of the bile.

In the natural state, these two substances are distinct; but yet a certain degree of attention is requisite to distinguish them. When more blood than usual happens to stagnate in the liver, the distinction between them is lost, and the organ presents a uniform red colour. When, on the other hand, it contains less blood than usual, the yellow substance becomes more apparent, and in some cases the deficiency of blood is so great, that the red substance loses its colour, and the whole of the liver presents a whitish tinge. 583.

These shades of colour may be owing to a mechanical obstruction to the venous circulation, in which case the liver is uniformly red—to the diminution of the total mass of the blood, blanching the organ—or to certain affections of the organ itself, which affect its circulation, and induce a state of hyperæmia or anæmia. Other changes may also take place. Thus the white substance may become hypertrophied, in two degrees—one, where the substance of the organ is traversed by lines or circumvolutions of a yellowish white colour, much more distinct than in the natural state—the other, where both the interior and exterior are studded with numerous granules, either isolated or agglomerated, and exactly resembling yellow wax. These whitish granules he considers to be merely the white substance in a state of hypertrophy.

"While the white substance of the liver is thus preternaturally developed, the red may continue in its natural condition, or may be altered in its colour, which often becomes very pale or olive green; and in its bulk, which may be either increased or diminished. Laennec remarks that *cirrhosis* is often accompanied by a shrivelled state of the liver. The red substance, as it wastes away, becomes infinitely less vascular, and, in certain cases, is in a great measure transformed into cellular or cellulo-fibrous tissue. This state of the liver is almost constantly accompanied by ascites." 586.

The red substance is also susceptible of a remarkable kind of hypertrophy which produces in the interior of the liver small, hard, red masses, which are distinguished from the surrounding parenchyma by their greater consistence and deeper colour. These may be unequal in form and size, or else distributed through the liver, so as to divide it into a number of similar lobules.

### II. LESIONS OF THE HEPATIC CIRCULATION.

Hyperæmia is considered by M. Andral as one of the most frequent morbid conditions of the liver. It is sometimes general, and the organ is then uniformly red—its volume increased—and its consistence little altered, when the affection is simple. In many cases the hyperæmia is only partial, forming red spots in different parts, surrounded by paler parenchyma.

"Hyperæmia of the liver is of three kinds.

The first results from a process of irritation. This irritation is sometimes idiopathic, and sometimes subsequent to a similar affection of the alimentary canal.

In the second kind, the blood accumulates in a manner wholly passive in the parenchyma of the organ, just as it does in the gums of scorbutic patients.

The third kind is purely mechanical, being observed where there is any obstacle to the free entrance of the blood into the right side of the heart; the blood then stagnates in the supra-hepatic veins, and *obstructs* the liver.

Congestion of the liver from a mechanical cause is frequently produced in infants while coming into the world; and such of them as die in a state of asphyxia have that organ so gorged with blood, that it sometimes ruptures its vessels and is effused in a layer on its convex surface beneath its investing membranes. M. Billard has repeatedly seen an effusion of blood into the abdomen produced by this turgid state of the liver.

Instead of accumulating in the hepatic capillaries, the blood may escape from its vessels, and become effused into the parenchyma of the organ, thus producing a kind of hepatic apoplexy. Some of these hæmorrhages are owing to the rupture of one of the principal vessels distributed to the liver.* In other cases, however, there is no perceptible rupture of any vessel, all that is observed being a collection of fluid or coagulated blood in one or more points of the liver. This was well exemplified in a liver shewn me by M. Rullier, which, besides various collections of fluid and semicoagulated blood, contained also some of a firmer consistence, in the centre of which were contained several hard fragments of fibrine deprived of their colouring matter. The examination of this liver led me to inquire whether fibrine thus deprived of its colour might not be the origin of certain accidental productions, such as encephaloid and others, that are often found in the liver; and my conjecture received additional confirmation from the examination of another liver shown me shortly after by M. Reynaud, in which I was able to trace the various changes of the blood from the perfectly fluid state until it passed into a substance possessing all the characters of encephaloid." 589.

### III. Lesions of Nutrition.

Hypertrophy and atrophy produce, of course, a change in the size of the organ; while other lesions induce softening or induration. Hypertrophy of the liver is of several kinds, as regards colour, consistence, and form. The colour is sometimes extremely pale—deep red, or variegated, with grey, green, brown, and black tints. The livers of the fœtus and of very young children are naturally in a state of hypertrophy, as compared with the adult. As the infant advances in age, the liver *diminishes* in size—ceases to extend into the abdomen, and retires behind the ribs, below which it does not extend, except when in a state of disease. In some cases, however, the child, and even the adult, retain this natural hypertrophy of infancy. This state is generally connected with other perversions of nutrition, forming altogether the scrofulous diathesis.

"Atrophy of the liver, considered as affecting its substance generally, may extend to the three lobes, or be confined to one, and may be accompanied by induration or by softening.

The liver, when in a state of atrophy, is generally diminished in size; this, however, is not necessarily the case, as it is sometimes as large as, or even larger than in the natural state; but then, in proportion as its proper tissue has disappeared, it has been replaced by cellular tissue. In such cases the organ having lost its peculiar structure and organization, is reduced to its pri-

* Vide *Clinique Medicale.*

mitive frame-work, and large patches are found in it occupied only by cellular tissue, which sometimes becomes hypertrophied, and in some cases contains serous cysts or hydatids, which, far from announcing an augmentation of the organic action of the part where they appear, are perhaps connected with its diminution; the cellular tissue, though unable to produce the hepatic parenchyma, showing its tendency to organization by becoming a serous cyst.

Induration of the liver has long attracted the attention of medical men. It is frequently accompanied by hypertrophy or atrophy of the parenchyma; but it may also exist without either. The liver, when indurated, may be of a lighter or deeper red, or of a grey, green, or brown colour.

Softening of the liver is, at least, as frequent as its induration. There are two degrees of it. In the first, the diminution of consistence of the parenchyma is not perceived until it is pressed between the fingers, when we find that it readily gives way, and is reduced to a pultaceous mass. In the second, which is much more uncommon, the softening is evident to the eye, the tissue of the organ presenting an appearance similar to that given it by prolonged maceration: the vascular apparatus is, in a manner, dissected from the cellular frame-work, and its ultimate branches, deprived of their uniting medium, float in a red or grey pulp, which seems to be merely the hepatic parenchyma reduced to the fluid state.

The softened liver sometimes retains its ordinary colour; in some cases, it is in a state of hyperæmia, and consequently red or brown; and in others, it is remarkably pale, which seems to result from its tissue being modified in such a manner as no longer to admit the colouring matter of the blood, of which there are no traces to be found except in the large vessels."* 593.

### IV. LESIONS OF THE SECRETIONS OF THE LIVER.

The liver is known to secrete a fatty matter, and if this be in too great quantity, it gives rise to certain morbid appearances in the organ.

" The secretion sometimes occupies the whole extent of the organ, and sometimes exists only in some scattered points. Instead of being infiltrated through the parenchyma, it is occasionally collected in some one spot, being deposited there like tubercle or pus, and forming grey or white morbid masses, which thrust back the substance of the liver, and present to the eye and the touch all the properties of fat. Masses like these have been found wholly formed of cholesterine. The causes that give rise to this fatty secretion in the liver are as yet unknown, it being a mere hypothesis to attribute it to irritation." 595.

It is curious that almost all the cases presenting the fatty degeneration, have been consumptive patients—that is, people in whom the blood was no longer properly elaborated, and in whom the pulmonary exhalation could not be accomplished as in the natural state. Hypotheses will not fail to be erected on this foundation.

Of abscesses in the liver there is nothing worthy of notice in this part of Andral's work. The able author says that hepatic abscesses are so rare in these climates that many authors have actually doubted their existence. This is carrying the thing rather too far. Unquestionably the affection is infinitely rare compared with what occurs in tropical climates; but still the disease is seen sufficiently often to dispel all doubt respecting its existence.

---

* Vid. *Clinique Médicale*, (Maladies de l'Abdomen.)

### Cancer of the Liver.

This designation is rarely if ever applied to morbid degenerations of the liver in England. It is but fair to hear what M. Andral has to say on the subject.

"Writers have described by the name of cancer of the liver, an alteration of that organ in which certain morbid productions distinguished by well marked physical characters are deposited in its parenchyma. They are those that have been described in the first volume by the names of *encaphaloid* and *colloid* matter. They produce in the liver masses of various sizes, that are sometimes uniformly white, and sometimes white mixed with red. Their consistence is not always the same, some of them being firmer than the surrounding parenchyma, and others resembling a greyish pap, in the midst of which a greater or less quantity of blood is often effused. These masses frequently occupy the greatest part of the organ, leaving scarcely a vestige of its natural tissue between them. They occasionally project on its external surface; and the liver then has a knobbed appearance which is sometimes perceptible through the abdominal parietes.

It follows from some facts already mentioned, that these so called cancerous masses may arise from an effusion of blood, which, when coagulated within the substance of the liver, undergoes the various changes I have described. But, it is far from being proved that such is always the origin of these cancerous tumours. In many cases, all that we can discover, is at first the infiltration of a minute portion of the parenchyma of the organ with a whitish matter, the parenchyma being at the same time more or less injected at the point of infiltration or around it. This whitish matter gradually becomes more abundant, and the proper tissue of the organ no longer displays such an appearance of vascularity, though its vessels may still be detected by dissection or maceration; and it is then often discovered that the vessels traversing the morbid mass, which at first appeared to be vessels of new formation, belong to the liver itself. The surrounding parenchyma generally falls into a state of atrophy, but it may also become irritated and inflamed, in which case it often secretes pus, or ulcerates, and in this way produces a communication between the mass of encephaloid and the cavity of the peritoneum or of the intestine." 601.

From the above it is evident that what is termed cancer of the liver by the French pathologists, is the fungus hæmatodes of British anatomists.

### Hydatids.

These are very common occurrences in the liver, and the cysts in which they are contained, are sometimes so enormous as to occupy almost the whole of the organ. The parietes of these cysts are generally composed of fibrous membrane, capable of being detached from the tissue of the liver without tearing it.

### V. Diseases of the Biliary Ducts.

The author sets out with the following passage.

"The biliary ducts and gall bladder are liable to various alterations, none of which give rise to any unpleasant symptoms during life, unless they produce a diminution of their caliber." 602.

M. Andral, of course, excludes morbid secretions, or formations, as biliary calculi, from diseases of the parts containing them. The lining mucous membrane of the biliary ducts, he observes, is sometimes so swollen, from the effects of hyperæmia, as to contract, or even totally obstruct the passage of the part affected. He has seen jaundice arise from this cause.

"When the ducts have been obliterated for a certain length of time, the gall bladder, which was at first dilated, contracts, the bile is absorbed out of it, and its diminished cavity contains only a little mucous matter, or else is completely filled with calculi.

Under the influence of acute or chronic irritation, the walls of the biliary ducts sometimes become softened or ulcerated, and eventually perforated; in consequence of which the bile escapes into the cavity of the peritoneum. The perforation occasionally takes place in some point behind where the duct is obstructed.

The gall bladder presents the same alterations as the ducts, its walls being found red, ulcerated, softened, or perforated. In this last case, the bile sometimes escapes into the peritoneum, and sometimes is evacuated through a perforation in the integuments." 604.

### VI.—Alterations of the Bile.

We cannot establish any connexion between the alterations of the liver and those of the bile secreted in it. In the majority of those organic diseases which have been described, the bile in the ducts and gall-bladder does not appear at all changed in quantity or quality. In other cases, again, where the structure of the liver does not appear at all changed, the bile is either excessive or deficient in quantity, or altered in its sensible qualities.

"I have sometimes been astonished at the immense quantity of this fluid in the intestines, in cases where they were but slightly inflamed, and the liver did not appear in the least altered.

The reason of the secreting fluid's being altered without the secreting organ's being so, is, that in the liver, as in every other organ destined to separate a fluid from the blood, the alterations of texture that are apparently the most serious, are not always those that exert the greatest influence over the act of secretion; the derangement of this secretion seems to depend rather on certain lesions of the organ that escape our observation, and not unfrequently on the lesions of other parts. Thus, Magendie's experiments have proved that, by changing the food of an animal, we can alter at pleasure the composition of the bile, which is evidently owing to the previous alteration of the blood from the same cause." 607.

Alterations in the quality of the bile may be discovered by simple inspection—by physiological experiments—and by chemical analysis.

"We have long been aware, from experiments on animals, that the bile taken from some dead bodies, produces no other inconvenience when introduced into the living body than an inconsiderable irritation, while that taken from others produces much more serious consequences, and sometimes even death itself. In some instances, it may be touched and tasted with perfect safety; in others, it produces pustules, ulcers, &c., on the tongue and lips. Here, then, we have very important changes in the bile, which we never could have learned from anatomy.

The only alterations in the quality of the bile discoverable by inspection are changes in its colour and consistence. It has been observed to have every shade of colour from the deepest black to an almost transparent whitish tint. Its consistence is also very variable, it being sometimes as thick as pitch, sometimes like glue, and sometimes fluid as water.

We learn from chemical analysis that the different component elements of the bile vary greatly in their proportions. Sometimes, especially in cases of fatty liver, is is found to contain scarcely any thing but water and albumen. At other times, the yellow matter, the resin, or the cholesterine is the predominant principle. The causes on which these variations depend are as yet unknown." 608.

It would be an interesting inquiry, and one far more profitable in practice, to endeavour to ascertain the phenomena produced in the human body by its own bile, in various states of sensible alteration. This part of the inquiry M. Andral has entirely overlooked. We have only room here to make one observation of a therapeutic nature. We recently saw two instances of the most obstinate and long-protracted jaundice, where the patients were reduced to skeletons, and the skin, for many months, the colour of mahogany; and where inspissated ox-gall produced the best effects—tinging the motions, lessening the irritability of the stomach, and increasing the peristaltic action of the intestines. In both instances recovery ultimately took place, after all hopes had been abandoned by physicians and friends.

With the following passage, containing a true statement, and not much calculated to render us very dogmatical in our prognoses and diagnoses, we shall conclude this article.

"It was supposed that jaundice always arose from some obstacle in the biliary ducts to the passage of the bile into the duodenum, but this opinion is incorrect, inasmuch as those ducts are often found perfectly free in persons that die of the disease. Indeed nothing can be more variable than the state of the liver in jaundice; any one of the numerous alterations to which that organ is subject, may be attended by it, but none of them are constantly or inseparably connected with it. There are even cases of icterus where we cannot discover any lesion whatever in the liver or its appendages; and in many such cases we have reason to doubt that the liver had any thing to do with the disease. We are not to suppose, however, that the yellow tinge of the skin can be produced only by the presence of the colouring matter of the bile in the blood, as it sometimes seems to arise merely from a sanguineous suffusion of its tissue. Such, especially, seems to be the nature of the *icterus neonatorum,* in which we can observe the red tinge of the skin gradually changing into a yellow, which in its turn fades away, and is succeeded by the natural colour of the part. Neither can we find in the liver of children that die of this disease any constant lesion that could account for it. Some have asserted, indeed, that in such cases they found the liver gorged with blood; but it is found at least as frequently in the same state where there has been no jaundice at all." 611.

